# The Difference of Chemical Components and Biological Activities of the Raw Products slices and the Wine Steam-Processed Product from Cistanche deserticola

**DOI:** 10.1155/2019/2167947

**Published:** 2019-03-17

**Authors:** Ying Zhang, Yuewu Wang, Song Yang, Yunfeng Xiao, Haibin Guan, Xin Yue, Xiaoqin Wang, Xiangri Li

**Affiliations:** ^1^School of Chinese Materia Medica, Beijing University of Chinese Medicine, Liangxiang Higher Education Park, Beijing 102488, China; ^2^School of Pharmacy, Inner Mongolia Medical University, Jinshan Development Zone, Hohhot, Inner Mongolia 010110, China; ^3^The Center for New Drug Safety Evaluation and Research of Inner Mongolia Medical University, Jinshan Development Zone, Hohhot, Inner Mongolia 010110, China

## Abstract

As a well-known Chinese herb medicine, the Cistanche deserticola has been used for the treatment of kidney deficiency syndrome in China for thousands of years. Both the raw product of Cistanche deserticola slices (RCD) and its Wine Steam-Processed Product (WSCD) are used clinically for different effects. In this study, the influences of steaming process with wine (SPW) from Cistanche deserticola on chemical compositions and biological effects were investigated. The principal component analysis (PCA) and quantitative analysis were used to study the differences of the chemical compositions. The effects of nourishing kidney were also investigated to compare the differences between the RCD and the WSCD. The PCA results indicated that the obvious separation was achieved in the RCD and WSCD. The results of quantitative analysis showed that the WSCD has higher amounts of total polysaccharides, total PhGs, isoacteoside, and osmanthuside B than RCD, while the content of 2′-acetylacteoside and acteoside decreased after the SPW. The comparison of RCD and WSCD on biological activities showed that both could restore the level of sex hormone in the model of kidney-yang deficiency and improve the antioxidant effect. The WSCD were much better in increasing the viscera weight of kidney and seminal vesicle. The results indicated that SPW changed its chemical components and enhanced its biological activities.

## 1. Introduction

*Cistanche deserticola* is a common traditional Chinese medicine and has been generally used as a tonic in China and Japan for many years, commonly known as “Desert ginseng.”* Cistanche deserticola* was first recorded as a top grade in “Shennong's Herbal Classic of Materia Medica (Shen Nong Ben Cao Jing)” in about 100 B.C and used to treat various diseases including kidney deficiency, impotence, female infertility, morbid leukorrhea, profuse metrorrhagia (whites), cold sensation in the loins and knees, and chronic constipation in the elderly [1]. So far, several main constituents have been isolated, such polysaccharides [2, 3], phenylethanoid glycosides (PhGs) [1, 4], iridoids [1] and lignanoids [1, 5]. Among them, PhGs and polysaccharides have been shown to be the main biologically active components in* Cistanche deserticola* [6–9]. Modern pharmacological experiments have proved that* Cistanche deserticola* can enhance the production of testosterone and protect sperm, stimulate cell proliferation and enhance the cell survival rate, exhibiting marked activities for sexual potency, improving memory, antiaging, free radical scavenging, and neuroprotection [10–13].

In order to reduce toxicity and/or enhance the effects, most of the traditional Chinese herbs should be processed before prescription. The traditional process steps include cleaning, water process (grinding in water, steaming, and roasting), and fire process (stir-heating with wine, vinegar, salt, or honey) [14]. During the process, the chemical components maybe changed: the relative contents of certain components may be changed or new components may be formed [15]. The Process of* Cistanche deserticola* has long history and* Cistanche deserticola* should be processed by soaking in the rice wine, steaming as described in Chinese pharmacopoeia (2015 edition) [16]. SPW has been proved to influence their effects on kidney meridian according to the theory of traditional Chinese medicine (TCM) [17]. WSCD is first documented in “Lei Gongs Treatise on Preparation and Boiling of Materia Medica (Lei Gong Pao Zhi Lun)” in the Northern and Southern Dynasties of China. WSCD is better used to treat kidney deficiencies and protect semen as recorded in “Taiping Shenghui Fang” in Song Dynasty of China. However, there were no comparative studies on the chemical components between RCD and WSCD so far.

Modern pharmacological investigations have indicated that WSCD could tonify the kidney to secure essence by stimulating the hypothalamus-pituitary-gonad axis with varying degrees and is used to the treatment of deficiency in the kidney such as deficiency of kidney-yang [18]. However, up to now, the differences of biological activities between RCD and WSCD have not been researched. Recently, to facilitate the experimental research, the kidney-yang deficiency animal model has been duplicated by injecting rats with a high dose of hydrocortisone, in which rats will show symptoms greatly resembling those described in kidney-yang deficiency of TCM [19, 20]. Rats with kidney-yang deficiency always have some symptoms such as weight loss, food consumption reduction, weakness, increased water intake, and decreased activity. Based on this animal model, the differences of nourishing-kidney effects between RCD and WSCD were detected and even to reveal the scientific essence of traditional Chinese process.

## 2. Materials and Methods

### 2.1. Materials and Chemicals

The standard substance such as acteoside, isoacteoside, echinacoside, cistanoside A, and 2′- acetylacteoside were purchased from Shanghai Yuanye Biotechnology Co. Ltd (Shanghai, China). Cistanoside F, cistanoside C, osmanthuside B, and tubuloside B were purchased from Chengdu Pfeide Biotechnology Co. Ltd (Chengdu, China). The purity of all standards was no less than 98%. HPLC grade methanol and acetonitrile was purchased from Aladdin Chemistry Inc. (Shanghai, China). Deionized water was obtained using a MilliQ50 SP Reagent Water System (Bedford, MA, USA) for preparing samples and mobile solution. All other organic solvents used in this study were of analytical grade and purchased from Shanghai Chemical Co. Ltd (Shanghai, China).

### 2.2. Samples Collection

All the raw* Cistanche deserticola* (7 batches of samples) were collected by Inner Mongolia Medical University from Inner Mongolia and Ningxia provinces. All were identified as* cistanche deserticola *Y. C. Ma by Xiao-qin Wang, the professor of Pharmacognosy Department in Inner Mongolia Medical University. A voucher specimen was deposited at the School of Pharmacy, Inner Mongolia Medical University. After collection, the inflorescences of* Cistanche deserticola* were removed, the stems were sliced and dried at room temperature with air, and then the slices were randomly divided into two groups in each batch: one is RCD and the other is used for the preparation of WSCD.

The WSCD were prepared in the lab according to Chinese pharmacopoeia (2015 edition) [16], which means that the sliced RCD were soaked in rice wine with a closed container for 6h until softness, steamed in a water bath for additional 12h until the surface becomes black, and dried at room temperature with air.

### 2.3. Principal Component Analysis (PCA)

Principal component analysis (PCA) is a sophisticated technique widely used for reducing the dimensions of multivariate problems. It reduces the dimensionality of the original data set by explaining the correlation among a large number of variables in terms of a smaller number of underlying factors without significant loss information. In this study, the differences between RCD and WSCD were performed by unsupervised PCA using the SIMCA 13.0 software based on the relative peak areas in the HPLC chromatography. With the help of PCA, the main chemicals' influences on the classification among different samples were found out.

### 2.4. Sample Preparation

#### 2.4.1. Extract Preparation for Animals

The RCD and WSCD were selected from the samples collected and prepared as described in Section 2.2.

Air-dried and sliced RCD was powdered by pulverizer (FW135, Tianjin Taisite Instrument Co., Ltd.), accurately weighed 1.0 kg, and soaked in 50% ethanol for 30 min, and the ratios of plant/ethanol used were 1/10(w/w). And then, it was extracted under reflux twice for 1 h each time. The two extracts were combined and filtered and the ethanol was recovered under the reduced pressure at 60°C. The total crude extract of PhGs was dried in vacuum at 60°C and purified by macroporous resin. Finally, the PhGs extract of RCD was obtained and accurately weighed.

The PhGs extract of WSCD was obtained following the same procedure above.

The sediments from extracting the PhGs were air-dried and decocted twice for 1.5 h each time with 20 times of water. The two extracts were combined and centrifuged at 4000 rpm for 10 min, and the supernatant was concentrated and precipitated with 95% ethanol. After centrifugation, the precipitate was dried in vacuum at 60°C. Finally, the polysaccharide extract of RCD was obtained and accurately weighed.

The polysaccharide extract of WSCD was obtained following the same procedure above.

The PhGs and polysaccharide extracts of RCD and WSCD were mixed suspension in water, respectively, when the rats were treated orally.

#### 2.4.2. Sample Preparation for the Determination of PhGs

The PhGs extracts in Section 2.4.1 were accurately weighed as 0.15 g and extracted by ultrasonication with 50mL 50% aqueous methanol solution for 40 min. After cooling, the loss of weight was replenished with 50% methanol. All samples and solvents were filtered through 0.45 *μ*m membrane before analysis. The content of four PhGs in RCD and WSCD was determined using HPLC, and the total PhGs was determined using UV spectrophotometry.

#### 2.4.3. Sample Preparation for the Determination of Polysaccharide

The polysaccharide extracts in Section 2.4.1 were accurately weighed as 0.10 g and extracted by ultrasonication with 50 mL hot water for 40 min; after cooling, the loss of weight was replenished with water. All samples and solvents were filtered through 0.45 *μ*m membrane before analysis.

### 2.5. Chromatographic Condition of HPLC and Determining the four PhGs

The chromatographic separation was performed in an UltiMate 3000 HPLC system (Thermo Fisher Scientific, USA), equipped with a dual-gradient pump, a column compartment, and a DAD detector. Data was collected and processed using a ChromeLeon Chromatography Data System. The samples were separated on an Agilent Zorbax SB-C18 (250mm×4.6 mm, 5 *μ*m) with a C18 guard column (4.6mm×12.5 mm, 5 *μ*m). The mobile phase consisted of acetonitrile (A) and 0.1% phosphoric acid solution (B) at a flow rate of 1 mL/min.The gradient elution is as follows: initial 0–13 min, linear change from A-B (5:95, v/v) to A-B (15:85, v/v); 13–25 min, linear change to A-B (20:80, v/v); 25–43 min, linear change to A-B (25:75, v/v). The wavelength of the detector was monitored at 330 nm. The column temperature was set at 30°C and the sample volume injected was 10 *μ*L [21]. The stock solution containing four reference standards was prepared by dissolving the reference standards in 50% methanol to a final concentration of 0.20 mg/mL for 2-acetylacteoside, 0.20 mg/mL for acteoside, 0.05 mg/mL for osmanthuside B, and 0.10 mg/mL for isoacteoside. The solution was then diluted to five different concentrations with three replicates to establish calibration curves. The sample content was expressed in g/kg of raw weight.

### 2.6. Determination of Total PhGs

The total PhGs was determined using UV spectrophotometry method at the wavelength of 330nm in UV1000 spectrophotometer (Shanghai Tianmei Scientific Instrument Co., Ltd.). The stock solution was prepared by dissolving echinacoside standards in 50% methanol to a final concentration of 0.10 mg/mL and then diluted to five different concentrations with three replicates to establish calibration curves.

### 2.7. Determination of Polysaccharides

The determination of polysaccharides was carried out using the phenol-sulfuric acid method. 1mL of the sample solution was put into 20 mL test tube with glass stopper, and 1 mL of 6% phenol solution and 5 mL of concentrated sulfuric acid were added and shook for 5 min. The mixture was transferred to a boiling water bath for 10 min and cooled to room temperature for ultraviolet detection. The ultraviolet absorption was monitored at 480 nm in a UV1000 spectrophotometer. The reference standard of anhydrous D-glucose was accurately weighted and dissolved in distilled water to a final concentration of 0.10 mg/ mL. Then the solution was diluted to five different concentrations with three replicates to establish calibration curves.

### 2.8. Animal Experiments

#### 2.8.1. Animals and Housing

Male SD rats of sexual maturity (180-200 g) were purchased from Xinglong (Beijing) Experimental Animal Farm (age: 6 weeks old) and the laboratory animal license was SCXK (Jing):2016-0003. All animal procedures were approved by the Inner Mongolia medical University Animals Research Committee and carried out according to National Institute of Health guidelines regarding the principles of animal care (2004). All animals were kept at a barrier system with regulated temperature of 21–23°C and relative humidity of 40–65% and on a 12 h dark/light cycle. The rats were given* ad libitum* food and water and acclimatized to the above environment for a week.

#### 2.8.2. Dosage and Sampling

The rats were transferred to individual metabolic cages and randomly divided into 6 groups (*n* = 10 in each group). They were given an intramuscular injection of 15 mg/kg hydrocortisone sodium succinate (purchased from Tianjin Biochemical Pharmaceutical Co., Ltd., Tianjin, China) for 2 weeks except for group 1 which were injected with an equal volume of physiological saline. On the 14th day, the body weight, food consumption, water intake, urine volume, and spontaneous activity within 5 minutes were collected to make sure whether the kidney-yang deficiency model was successfully made. From the 15th day, 6 groups were treated as follows: group 1 and group 2 (model group, M) were treated with equal volume of distilled water, group 3 (PhGs extract of RCD group, PR) were treated with 0.42 g/kg PhGs extract of RCD, which was equal to about 1.8 g/kg of the raw* Cistanche deserticola* and the other groups were the same, group 4 (PhGs extract of WSCD group, PW) were treated a dose of 0.49 g/kg PhGs extract of WSCD, group 5 (polysaccharide extract of RCD group, SR) were treated with 0.18 g/kg the polysaccharide extract of RCD, and group 6 was (polysaccharide extract of WSCD group, SW) were treated a dose of 0.22 g/kg the polysaccharide extract of WSCD. All rats were treated by gastric perfusion per day. After a month's treatment, the rats were denied food for 12h before blood collection. On the next day, all rats were anesthetized and sacrificed. During the testing, the rats were weighed once a week for adjusting the dosage and weighed again before sacrificing. Blood samples were collected in Eppendorf tube with 10% EDTA-2Na solution. The serum was separated via centrifugation at 2000 rpm for 15 min and stored at -80°Cfor further use. In addition, kidneys, testicles, epididymis, prostate gland, and seminal vesicle were removed and weighed rapidly. After weighing, the testicles were frozen in liquid nitrogen for the determination of SOD and MDA.

### 2.9. Hormone and Antioxidant Effect Analysis

The testosterone (T) and estradiol (E_2_) level were determined in the Qingdao Kechuang Quality Inspection Center by radioimmunity and colorimetric method. The frozen testicles were weighed and mixed with 20 times of cold physiological saline (W/W). The testicle homogenate obtained by tissue homogenate in the ice bath was centrifuged to obtain the supernatant. The contents of SOD and MDA of testicle were determined by SOD and MDA kits following the instructions.

### 2.10. Statistical Analysis

The significance of differences among groups was compared through one-way ANOVA test followed by Scheffe's test with a significance limit of 0.05 using the SPSS software 25.0. All data was expressed as the mean ± standard deviation (SD) (n=3).

## 3. Results

### 3.1. The Analysis of PhGs Changes after SPW Using PCA

**Figure 1:**
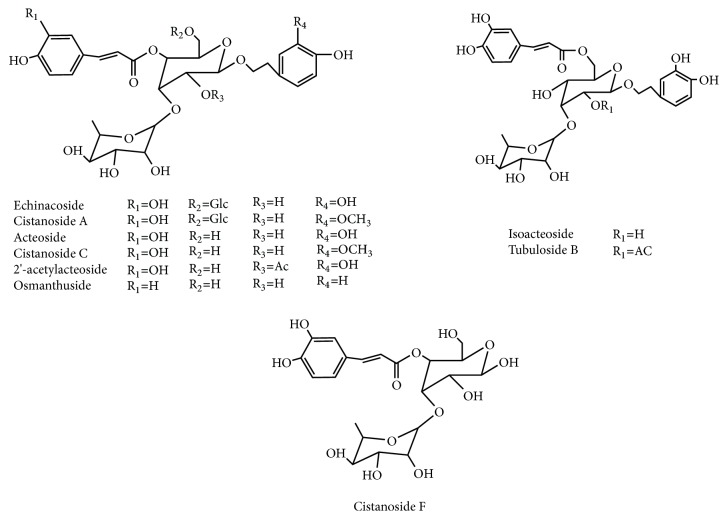
Chemical structures of the PhGs in* Cistanche deserticola *Y. C. Ma.

**Figure 2:**
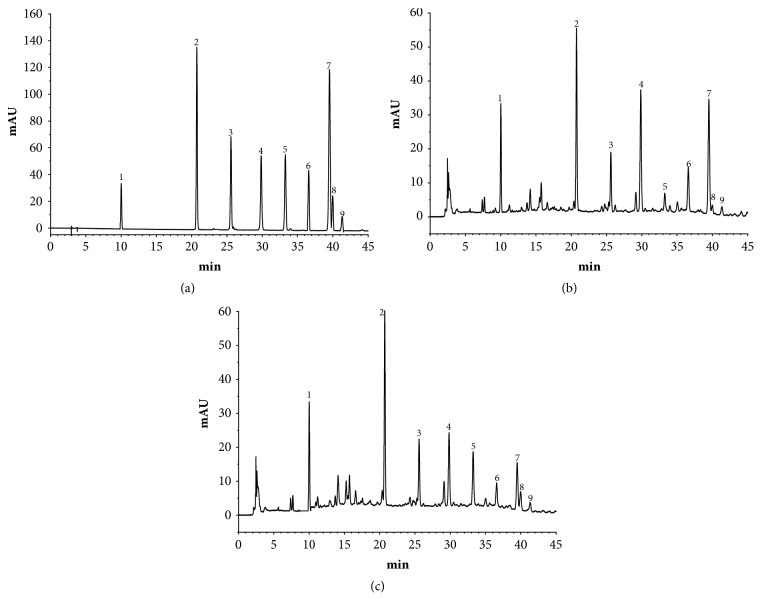
HPLC chromatogram of standard substance (a), RCD (b) and WSCD (c). Peaks: (1) cistanoside F, (2) echinacoside, (3) cistanoside A, (4) acteoside, (5) isoacteoside, (6) cistanoside C, (7) 2′-acetylacteoside, (8) osmanthuside B, and (9) tubuloside B.

**Figure 3:**
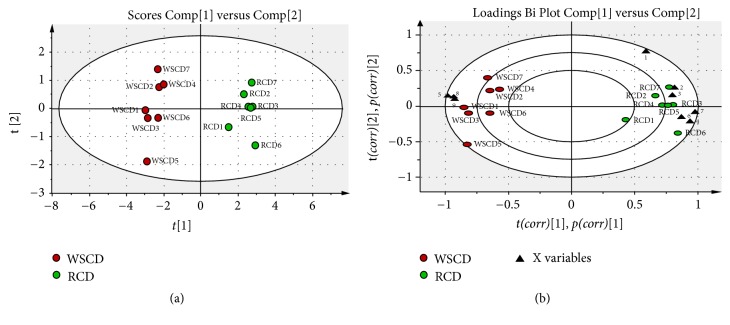
PCA score plot (a) and loading Bi plot (b) of RCD (green circle) and WSCD (red circle). RCD and WSCD were classified into two clusters. Peaks 2, 3, 4, 6, and 7 are the most important components to distinguish RCD and WSCD, which will reduce during SPW; peaks 5, 8 and 9 are also the most important components for the differences between RCD and WSCD, which will increase during SPW.

To compare the PhGs Changes after SPW, 9 chromatographic peaks were selected as characteristic peaks and were identified. Their structures were shown in Figure 1, the relative peak areas of which were calculated for quantitative expression. The HPLC chromatogram was shown in Figure 2, which indicated that the relative content of main PhGs was changed during SPW. PCA analysis on the relative peak areas of 9 components was obtained for discrimination of different samples. The RCD and WSCD were far from each other in the plot of the scores (Figure 3(a)), which indicated that the samples were classified into two clusters. So it is believed that the contents of the chemical constituents were different. To further find the potential chemical markers for the discrimination, the extended statistical analysis was performed to generate the loading Bi plot (Figure 3(b)). Peaks 2, 3, 4, 6, and 7 will decrease after SPW while Peaks 5, 8, and 9 will increase, and all of them were the most important components to distinguish the RCD and WSCD. The relative contents of peak 1 are not varied much after SPW.

### 3.2. The Contents of Total Polysaccharides and PhGs Changed after SPW

As shown in Figure 3(b), 8 components (5, 8, and 9 increased and 2, 3, 4, 6, and 7 decreased) had changed significantly and had a major impact on the sample clustering. Among them, isoacteoside and Osmanthuside B had increased the most while 2′-acetylacteoside and acteoside decreased the most. So, the contents of isoacteoside, Osmanthuside B, 2′-acetylacteoside and acteoside were determined by HPLC. As shown in Figure 1, peak 4 was acteoside, peak 5 was isoacteoside, peak 7 was 2′-acetylacteoside, and peak 8 was osmanthuside B. The total PhGs in RCD and WSCD were determined using UV method and the total polysaccharides were carried out using the phenol-sulfuric acid method, the results were shown in Table 1, isoacteoside, Osmanthuside B, total polysaccharides and PhGs in WSCD had increased significantly compared with RCD. 2′-acetylacteoside and acteoside had decreased significantly.

### 3.3. The Hydrocortisone-Induced Kidney-Yang Deficiency Model Was Made

**Figure 4:**
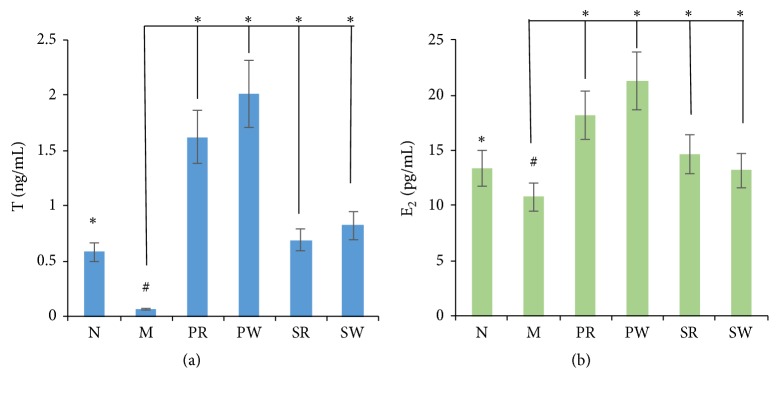
The levels of T and E_2_: (a) the level of T and (b) the level of E_2_. Significant differences with M group were designated as ^∗^*P* < 0.05. Significant differences with N group were designated as ^#^*P* < 0.05.

Compared with normal group, after 2 weeks' intramuscular injection of 15 mg/kg hydrocortisone sodium succinate, the body weight, food consumption, and spontaneous activity were significantly decreased (P<0.05) and the water intake and urine volume were increased; the results were shown in Table 2. The T and E_2_ levels in the serum of the model group were lower than normal group as shown in Figure 4. From the above, the kidney-yang deficiency model was successfully made.

### 3.4. The Impact of Extracts on the Average Viscera Weight and Index

All rats and their viscera were weighed and then the average viscera index was calculated.

Compared with the N group, the M group had significantly decreased the viscera weights of kidney, seminal vesicle, epididymis, and testicle, which also indicated that the hydrocortisone-induced kidney-yang deficiency model was successfully made. Compared with the M group, all observed viscera weight had increased in PW and SR group, the PR group had increased weight of testicle, epididymis, seminal vesicle, and prostate gland, and the SW group had increased weight of kidney, testicle, epididymis, and seminal vesicle. Compared with the PR groups, the PW group was better in kidney, seminal vesicle, and prostate gland. There was no significant difference between the SR and the SW groups in the weight of testicle, epididymis, seminal vesicle, and prostate gland. The results were shown in Table 3.

Compared with the N group, the viscera indexes of kidney, testicle, epididymis and seminal vesicle were decreased in M group. Compared with the M group, the PW and SR group had increased the index of kidney, testicle, prostate gland and seminal vesicle, the PR groups had increased the index of testicle, prostate gland and seminal vesicle, and the SW group had increased the index of kidney, testicle and seminal vesicle. Compared with the PR groups, the PW group was better in kidney, seminal vesicle and prostate gland. The SW and SR group had the similar index of kidney, testicle, seminal vesicle and epididymis. The results were shown in Table 4.

### 3.5. The Impact of Extract on the Level of Hormone (T and *E*_2_)

As shown in Figure 4, compared with M group, the levels of T and E_2_ in all the treatment groups (PW, PR, SW, and SR group) had increased significantly (P<0.05). However, the PW group was better than PR group in the level T and E_2_. The SR and SW groups had no significantly difference.

### 3.6. The Impact of Extracts on Antioxidant Effect

**Figure 5:**
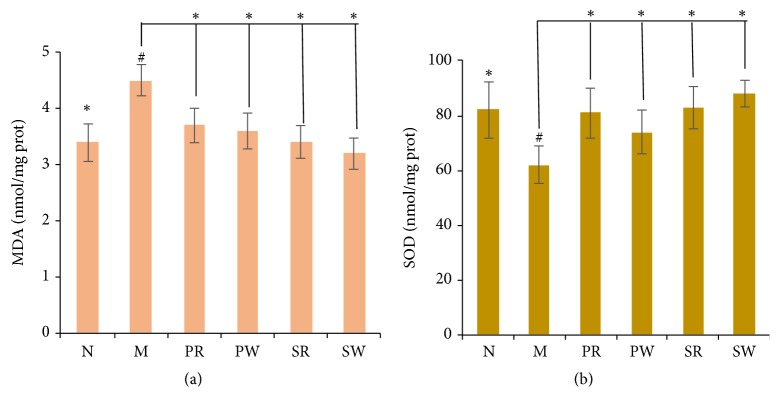
The contents of MDA and SOD: (a) the content of MDA and (b) the content of SOD. Significant differences with M group were designated as ^∗^*P* < 0.05. Significant differences with N group were designated as ^#^*P* < 0.05.

SOD is an important antioxidant enzyme. MDA is the product of lipid peroxidation which is the indicator of reflecting the degree of oxidant damage. The contents of SOD and MDA reflect the extent of oxidant and antioxidant ability. As shown as in Figure 5, Compared with M group, the antioxidant effect in all the treatment groups (PW, PR, SW, and SR) had been enhanced. The SW group had the greatest antioxidant effect.

## 4. Discussion

**Figure 6:**
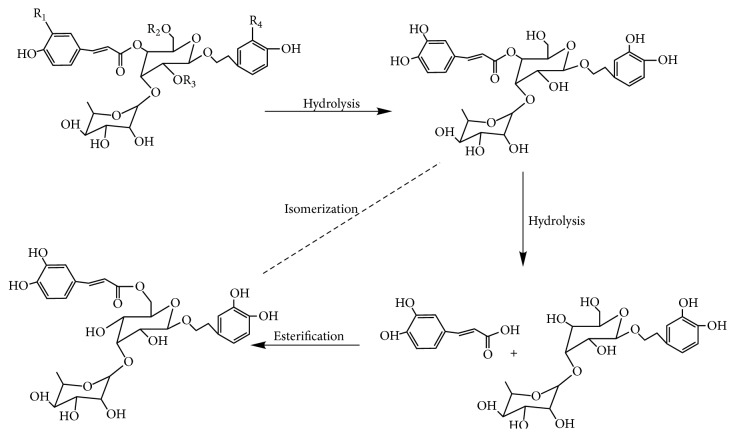
Proposed transformation pathways of the PhGs in* Cistanche deserticola* Y. C. Ma during SPW.

Before clinical application, the crude materials should be subjected to traditional Chinese process techniques. Steaming is one of traditional processing approaches for some Chinese medicinal herbs, which gives a black color produced by Maillard reaction [22], increasing the amounts of some bioactive components [23] and pharmacological activities. WSCD as one of the process products is better in terms of nourishing kidney compared with RCD as documented in the 2015 edition of Chinese pharmacopoeia. In this study, the PCA results indicated that the SPW had change the chemical profile of RCD. What is more, 8 components in PhGs had changed significantly. Total PhGs, total polysaccharides, isoacteoside, and Osmanthuside B had increased most, while 2′-acetylacteoside and acteoside decreased after SPW. The results indicated that SPW could change chemical constituents of RCD. The contents of PhGs with 1,3,4-trisubstituted glucopyranosyl moieties (such as acteoside, 2′-acetylacteoside, and cistanoside C) decreased in WSCD, however, the concentrations of their isomers with 1,3,5-trisubstituted glucopyranosyl moiety (such as isoacteoside and tubuloside B) increased, which indicated that the transformation of chemical constituents maybe occurred during SPW, and hydrolysis reaction will be one of the reasons. The proposed transformation pathways were shown in Figure 6. The increase in polysaccharides after SPW had also been reported in ginseng [24].

*Cistanche deserticola* was documented to invigorate the kidney and reverse the reduction in testosterone level and possess antioxidant effect and anti-inflammatory action [25], and PhGs and polysaccharides were the two main biologically active components in* Cistanche deserticola*. As PhGs, the isoacteoside and Osmanthuside B had antioxidation and anti-inflammatory effects [26, 27]. So, the comparison of pharmacological effects in testosterone level and antioxidant effect of RCD and WSCD was studied. In the model of hydrocortisone-induced kidney-yang deficiency, the endocrine was disrupted and the level of sex hormone was greatly decreased in the blood. In this study, the results of sex hormone analysis showed that both RCD and WSCD could significantly increase the level of T and E_2_, and both PhGs and polysaccharides showed the improvement of sex hormones. The WSCD was better than the RCD, especially in PhGs extract groups. The results of the viscera indexes showed that both PhGs and polysaccharides can improve the viscera weights and their weights indexes. In PhGs extract groups, the WSCD is better than RCD in increasing the weights and indexes of kidney and seminal vesicle. In addition,* Cistanche deserticola* has a significant antioxidant effect, especially the polysaccharides extract groups with WSCD. As discussed above, both RCD and WSCD could improve the kidney-yang deficiency syndrome; however, WSCD is better than RCD in increasing the level of T, E_2_, the weights, and indexes of epididymis and testicle. The results of the pharmacological effects study indicated that SPW had changed the chemical components of PhGs and increased the content of polysaccharides, which in turn increased the sex hormone level and enhanced antioxidant effect.

## 5. Conclusion

The study was to discover the change of PhGs, total polysaccharides, and the pharmacological effect in RCD and WSCD on the kidney-yang deficiency rat, especially on hormone level and antioxidant effect. This study has demonstrated that the process of traditional Chinese herbs may alter its chemical constituents and affect its bioactivity. It is also supported that raw products and processed products were prescribed differently in clinic.

## Figures and Tables

**Table 1:** The contents of polysaccharides and PhGs in RCD and WSCD (mg/g) (n=3).

	Total polysaccharides	PhGs	Isoacteoside	Osmanthuside B	2′-acetylacteoside	Acteoside

RCD	10.179±0.483	23.172±0.083	0.742±0.018	0.291±0.011	3.780±0.042	4.128±0.029
WSCD	12.381±0.579^∗^	27.457±0.079^∗^	2.259±0.046^∗^	0.884±0.023^∗^	2.085±0.033^∗^	3.076±0.028^∗^

*Note.* Significant differences with RCD were designated as ^*∗*^*P* < 0.05.

**Table 2:** The indicators changed after intramuscular injection of hydrocortisone sodium succinate, (n=10).

	N	M	PR	PW	SR	SW

Body Weight (g)	285.14±9.26	226.67±12.14^#^	237.89±16.87^#^	214.16±17.75^#^	231.57±14.33^#^	219.64±16.60^#^
Food consumption (g/24h)	16.27±3.24	12.39±4.86^#^	11.19±3.91^#^	12.62±2.79^#^	11.97±5.01^#^	13.17±4.28^#^
Water intake (mL/24h)	22.14±4.36	32.17±5.73^#^	34.24±4.22^#^	28.18±3.79	31.18±5.62^#^	33.04±7.14^#^
Urine volume (mL/24h)	10.17±3.01	16.91±5.81^#^	17.82±3.77^#^	15.34±4.15^#^	15.90±5.44^#^	16.34±3.98^#^
Spontaneous activity(n/5min)	100.4±14.72	72.7±21.59^#^	68.9±17.96^#^	62.8±14.91^#^	75.7±19.20^#^	70.3±23.27^#^

*Note.* Significant differences with N group were designated as ^#^*P* < 0.05.

**Table 3:** The viscera weight (g) (n=10).

	N	M	PR	PW	SR	SW

Kidney	2.8726±0.1104^∗^	2.5130±0.0836^#^	2.7528±0.0767	3.4331±0.0860^∗^	3.1734±0.1542^∗^	3.2372±0.1200^∗^
Testicle	3.2241±0.1098^∗^	2.8937±0.1248^#^	3.1492±0.0870^∗^	3.4007±0.1655^∗^	3.1543±0.1262^∗^	3.1362±0.1467^∗^
Epididymis	1.0747±0.0579^∗^	0.8787±0.0961^#^	0.9606±0.0402^∗^	1.0232±0.0802^∗^	1.0008±0.0992^∗^	1.0223±0.0751^∗^
Seminal vesicle	1.0002±0.0486^∗^	0.8840±0.0892^#^	0.8340±0.0218	1.0478±0.0925^∗^	1.0652±0.0557^∗^	1.0928±0.0387^∗^
Prostate gland	0.5279±0.0551	0.4793±0.0743	0.5681±0.0621^∗^	0.6417±0.0233^∗^	0.5599±0.0458^∗^	0.4884±0.0691

*Note.* Significant differences with M group were designated as ^∗^*P* < 0.05. Significant differences with N group were designated as ^#^*P* < 0.05.

**Table 4:** The average viscera index (g∕100g) (n=10).

	N	M	PR	PW	SR	SW

Kidney	1.0296±0.0609^∗^	0.9412±0.0313^#^	0.9938±0.0426	1.1962±0.0461^∗^	1.1624±0.0869^∗^	1.1456±0.0657^∗^
Testicle	1.1556±0.0787^∗^	1.0838±0.0719^#^	1.1369±0.0483^∗^	1.1849±0.0887^∗^	1.1556±0.0711^∗^	1.1832±0.0803^∗^
Epididymis	0.3852±0.0319^∗^	0.3291±0.0554^#^	0.3683±0.0223	0.3565±0.0430	0.3666±0.0559	0.3638±0.0411
Seminal vesicle	0.3585±0.0268^∗^	0.3311±0.0514^#^	0.3018±0.0121^∗^	0.3651±0.0496^∗^	0.3902±0.0314^∗^	0.3889±0.0212^∗^
Prostate gland	0.1892±0.0304	0.1795±0.0428	0.2051±0.0345^∗^	0.2236±0.0125^∗^	0.2051±0.0258^∗^	0.1738±0.03783

*Note.* Significant differences with M group were designated as ^∗^*P* < 0.05. Significant differences with N group were designated as ^#^*P* < 0.05.

## Data Availability

The data used to support the findings of this study are available from the corresponding author upon request.
